# Self-Interference Channel Training for Full-Duplex Massive MIMO Systems

**DOI:** 10.3390/s21093250

**Published:** 2021-05-07

**Authors:** Taehyoung Kim, Kyungsik Min, Sangjoon Park

**Affiliations:** 1 Department of Electrical and Electronic Engineering, Yonsei University, Seoul 03722, Korea; khotdog86@gmail.com; 2 Samsung Electronics Company Ltd., Suwon 16677, Korea; minkyungsik@gmail.com; 3 Department of Electronic Engineering, Kyonggi University, Suwon 16227, Korea

**Keywords:** full-duplex, massive MIMO, self-interference, channel estimation, partial training

## Abstract

Full-duplex (FD) is a promising technology for increasing the spectral efficiency of next-generation wireless communication systems. A major technical challenge in enabling FD in a real network is to remove the self-interference (SI) caused by simultaneous transmission and reception at the transceiver, and the SI cancellation performance depends significantly on the estimation accuracy of the SI channel. In this study, we proposed a novel partial SI channel training method for minimizing the residual SI power for FD massive multiple-input multiple-output (MIMO) systems. Based on an SI channel training framework under a limited training overhead, using the proposed scheme, the BS estimates only a part of the SI channel vectors, while skipping the channel training for the other remaining SI channel vectors by using their last estimates. With this partial training framework, the proposed scheme finds the optimal partial SI channel training strategy for pilot allocation to minimize the expected residual SI power, considering the time-varying Rician fading channel model for the SI channel. Therefore, the proposed scheme can improve the sum-rate performance compared with other simple partial training schemes for FD massive MIMO systems under a limited training overhead. Numerical results confirm the effectiveness of the proposed scheme for FD massive MIMO systems compared with the full training scheme, as well as other partial training schemes.

## 1. Introduction

Fifth-generation (5G) wireless communication systems, called new radio (NR), have been successfully commercialized at a global level [1,2]. The 5G NR provides a wide range of services with various requirements such as enhanced mobile broadband (eMBB), ultra-reliable low-latency communications (URLLC), and massive machine-type communications (mMTC) [3]. Despite the remarkable improvement in 5G NR in terms of performance and functionalities, there are extensive ongoing studies and standardization efforts to shape next-generation wireless communication systems, namely the sixth-generation (6G) [4]. It is expected that 6G will provide an ultimate experience beyond even that of 5G NR by enabling new services and applications such as multisensory extended reality, mobile holograms, connected robotics, autonomous systems, and wireless brain-computer interactions [5,6,7]. To meet the explosive data traffic expected in the 6G era, massive multiple-input multiple-output (MIMO) [8,9,10,11,12,13] and full-duplex (FD) [14,15,16] are indispensable technologies for improving the network capacity.

In massive MIMO systems, the base station (BS) is equipped with a large number of antennas, and the spectral efficiency can be significantly increased by simultaneously serving frequency resources and a large amount of user equipment (UE) concurrently [8,9,10,11,12,13]. Meanwhile, in FD systems, downlink (DL) and uplink (UL) transmissions occur simultaneously at the transceiver at the same time and frequency resource [14,15]. Thus, FD systems can theoretically double the spectral efficiency compared with conventional half-duplex (HD) systems such as time division duplex (TDD) and frequency division duplex (FDD) [16]. Therefore, the joint utilization of FD and massive MIMO can significantly improve the system capacity.

The main challenge for FD systems is the self-interference (SI) phenomenon, in which the signal transmitted from the BS or UE becomes an unwanted interference to the transmitter [17]. In the case of FD-BS, unattenuated DL signals incur SI for UL signals at the BS, where the power of the SI is significantly larger than that of the UL signals attenuated by the path loss. Therefore, to make the FD technique feasible, extensive studies have been conducted on SI cancellation (SIC) schemes. In general, both analog-domain SIC [18,19,20,21,22,23] and digital-domain SIC [24,25,26,27,28] are required. Regarding analog SIC schemes, radio frequency (RF) and baseband (BB) tapping approaches were studied in [18,19], respectively. Furthermore, a two-stage cancellation architecture that combines RF and BB tapping approaches was proposed in [20,21]. In [22], a time-varying least mean square (LMS) adaptive filtering scheme with step-size parameters decreasing with time was developed. In [23], a practical structure for an analog LMS (ALMS)-based analog SIC scheme was investigated. Regarding digital SIC schemes, the removal of both the fundamental and harmonic components of the SI based on the least square (LS) estimation of an SI channel was investigated in [24]. In [25], a digital SIC scheme that eliminates all transmitter impairments to mitigate the receiver phase noise and nonlinearity effects was proposed. Furthermore, in [26], a hybrid beamforming-based SIC scheme was proposed for FD MIMO systems in millimeter-wave communications. In [27], a digital SIC scheme based on an independent component analysis was proposed. In [28], an iterative nonlinear method was studied for FD systems under mixer imbalance and amplifier nonlinearity.

The level of SI cancellation and suppression depends highly on the estimation accuracy of the SI channel [29,30,31]. Therefore, improving the SI channel estimation accuracy is a key technical issue for FD systems, and several related studies have been conducted [32,33,34,35,36]. In [32,33], a joint estimation of the SI and data channels based on the maximum-likelihood (ML) approach was studied. In [34], a frequency-domain LS channel estimator was proposed, and an optimal pilot pattern was derived to minimize the sum of the mean squared error (MSE). In [35,36], channel estimation methods for FD systems with large-scale antennas were investigated.

Despite such studies on SI channel estimation, little effort has been made to investigate the SI channel training strategy when considering the training overhead problem for FD massive MIMO systems. Unfortunately, prior works such as [35,36,37,38] focused on the full training strategy for the SI channel estimation where all elements of the entire SI channel matrix are estimated. For the FD-BS, the size of the SI channel matrix at the BS increases with the number of BS antennas. In other words, assuming orthogonal training for SI channel estimation, the full training strategy requires the training overhead to linearly increase with the number of BS antennas [39,40]. As a result, a large training overhead is required for full SI channel training in FD massive MIMO systems. This not only decreases the duration of the data transmission phase in each transmission block for a potential spectral efficiency reduction, but also delays the beginning of the data transmission phase in each transmission block, which can be a crucial problem in latency-sensitive services such as URLLC. Therefore, the amount of training overhead for the SI channel can be strictly limited according to the system environment and services, which makes it impossible to apply the full SI channel training strategy. In this case, the BS can simply choose only a part of the SI channel vectors for training in either a random or round-robin manner under a limited training overhead. For random training, the BS arbitrarily chooses a part of the antennas for SI channel training at a given time instance, whereas for the round-robin training, the BS antenna is sequentially selected in a round-robin manner according to previous selections. However, because no optimization is considered for allocation, both partial training strategies can yield poor throughput performance. Thus, an optimized partial SI channel training strategy needs to be developed instead of an infeasible full training strategy and non-optimized partial training strategies.

Therefore, in this study, we propose an efficient SI channel estimation framework based on a partial training strategy for FD massive MIMO systems. In the proposed scheme, the pilot signals for SI channel training are allocated to a number of massive BS antennas to satisfy the limited training overhead constraint, and only a part of the SI channel vectors among the entire SI channel matrix corresponding to the allocated BS antennas is estimated for each transmission block. Considering that the SI channel can be modeled as a slowly varying Rician fading channel [17,30,38,41,42], we formulated an optimization problem to find an optimal pilot allocation strategy that minimizes the residual SI power after the SIC operation for a given Rician fading channel model. For this purpose, a closed-form expression for the residual SI power after the SIC operation was derived in terms of pilot resource allocation, and a comprehensive algorithm to solve the optimization problem was developed.

The contributions of this study are summarized as follows:

- We developed an efficient partial SI channel training framework for FD massive MIMO under limited training overhead. Considering that the SI channel can be modeled as a quasi-static channel, it is possible to reduce the training overhead by estimating only some of the SI channel vectors corresponding to a set of selected BS antennas, whereas the previous estimates are utilized for the SI channel vectors corresponding to the unselected BS antennas. This can improve the effective throughput in FD massive MIMO systems for a given training overhead.
- To find an optimal training strategy under the proposed SI channel training framework, we formulated an optimization problem to minimize the expected residual SI power after the SIC operation. To this end, we analyzed and derived a closed-form expression of the expected residual SI power in terms of pilot allocation. Based on the reformulated optimization problem, a simple algorithm to find the optimal allocation was applied, where the optimal allocation can be simply conducted through the BS antenna selection and pilot resource allocations.
- The effectiveness of the proposed scheme was verified based on extensive numerical results. It is shown that the proposed scheme can improve the UL sum-rate by approximately 116.7% and 57.8% at a maximum compared with the cases in which the pilot signals for the SI channel training are allocated randomly or in a round-robin manner, respectively. In addition, because the required training overhead for the SI channel training to achieve a given target UL sum-rate is decreased, the duration of the simultaneous DL and UL data transmission phase in each transmission block can be increased using the proposed scheme. As a result, for a given target UL sum-rate, the proposed scheme can also improve the DL sum-rate by approximately 13.2% at a maximum compared with the round-robin training.

The remainder of this paper is organized as follows. Section 2 describes the system model, including the channel model and basic communication procedure for FD massive MIMO systems. Section 3 presents the optimization problem for minimizing the residual self-interference power and provides a simple algorithm for training optimization based on joint antenna selection and pilot allocation. Section 4 provides numerical results to verify the effectiveness of the proposed method compared with other partial training methods. Finally, Section 5 concludes the paper.

Notations: In this paper, matrices and vectors are denoted in bold upper and lower cases, respectively. In addition, ${\left(\cdot \right)}^{*}$ and ${\left(\cdot \right)}^{H}$ denote the conjugate and conjugate-transpose operations, respectively; $x_{i}$ and $x_{ij}$ denote the *i*th column vector and the (i,j)th element of X, and $E\left[\cdot \right]$ denotes the expectation operator. Moreover, $\mathrm{tr}\left\{\cdot \right\}$ is the trace operator. $I_{N}$ is an N×N identity matrix. ${\left[I_{N}\right]}_{n}$ is the *n*th column vector of $I_{N}$. $\left|S\right|$ indicates the cardinality of the set S, and diag{·} and $\mathrm{sort}\left\{\cdot \right\}$ represent the diagonalization and ordering operations, respectively. CN(0,1) represents a complex Gaussian random variable with zero mean and unit variance.

## 2. System Model

**Figure 1:**
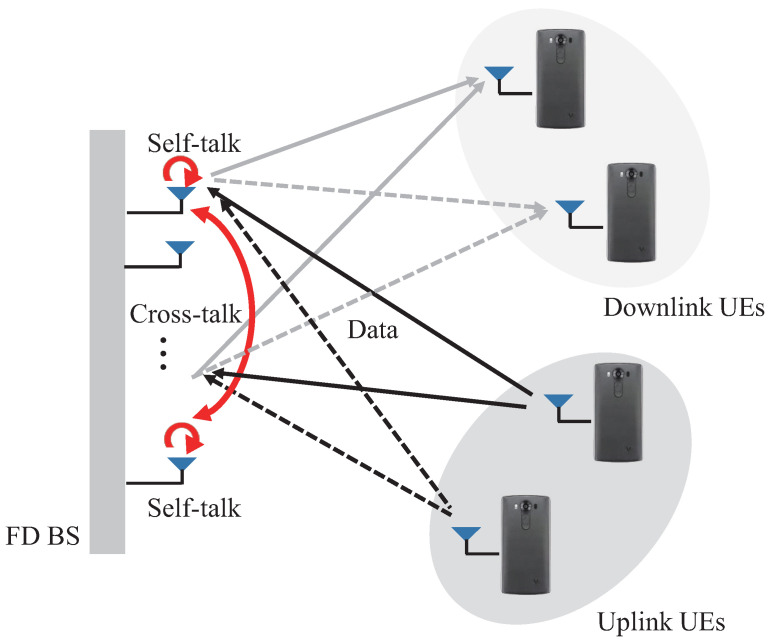
FD massive MIMO system model.

We considered a unidirectional FD system consisting of an FD-BS and HD-UE, as shown in Figure 1 [17]. It was assumed that the FD-BS is equipped with *M* shared antennas, such that the SI is composed of self-talk and cross-talk [14]. We considered $K(=K_{u}+K_{d})$ HD-UEs with a single antenna in which there are $K_{u}$ UL and $K_{d}$ DL UEs in the system.

A block-fading channel model was considered, where the channel was assumed to be static during a coherence block of length *T* channel uses [40]. We denote $H^{u}=\left[h_{1}^{u},\ldots ,h_{K_{u}}^{u}\right]$, $H^{d}=\left[h_{1}^{d},\ldots ,h_{K_{d}}^{d}\right]$, and $H^{s}=\left[h_{1}^{s},\ldots ,h_{M}^{s}\right]$ as an $M\times K_{u}$ UL channel matrix from the UEs to the BS, an $M\times K_{d}$ DL channel matrix from the BS to the UEs, and an M×M SI channel matrix at the BS, respectively. In addition, $h_{k}^{u}$, $h_{k}^{d}$, and $h_{m}^{s}$ are the channel vectors for the *k*th UL UE, the channel vector for the *k*th DL UE, and the SI channel vector from the *m*th BS antenna element to all BS antennas, respectively. The desired data signal channels, $H^{u}$ and $H^{d}$, are modeled as Rayleigh fading, that is $h_{ij}^{u}∼CN\left(0,1\right)$ and $h_{ij}^{d}∼CN\left(0,1\right)$, where $h_{ij}^{u}$ and $h_{ij}^{d}$ are the $\left(i,j\right)$ elements of $H^{u}$ and $H^{d}$, respectively. Moreover, $H^{u}$ and $H^{d}$ vary independently between two consecutive blocks. The SI channel matrix $H^{s}$ is typically modeled as Rician fading [17,30,38,41,42]. The SI channel is composed of two parts: (i) a strong near-field SI channel representing line-of-sight (LOS) paths and (ii) a weak far-field SI channel representing the reflected non-line-of-sight (NLOS) paths [38,41]. In addition, the NLOS paths are typically slowly changed because the BS is deployed at a high position and does not move. With the Rician fading model, the SI channel element $h_{ij}^{s}$ is given by the following:

(1) $$h_{ij}^{s}=\sqrt{\frac{\kappa_{ij}}{\kappa_{ij}+1}}{\bar{h}}_{ij}^{s}+\sqrt{\frac{1}{\kappa_{ij}+1}}{\tilde{h}}_{ij}^{s},$$

where $\kappa_{ij}$ is the Rician *K* factor, ${\bar{h}}_{ij}^{s}$ is the deterministic part with $|{\bar{h}}_{ij}^{s}{|}^{2}=1$, and ${\tilde{h}}_{ij}^{s}$ is a random variable that follows the Rayleigh distribution as ${\tilde{h}}_{ij}^{s}∼CN\left(0,1\right)$. It is assumed that ${\tilde{h}}_{ij}^{s}$ varies between two consecutive blocks, with the following correlation:

(2) $${\tilde{h}}_{ij}^{s}\left(n\right)=\sqrt{{\left(c_{ij}\right)}^{n^{\prime }}}{\tilde{h}}_{ij}^{s}\left(n-n^{\prime }\right)+\sqrt{1-{\left(c_{ij}\right)}^{n^{\prime }}}q_{ij},$$

where ${\tilde{h}}_{ij}^{s}\left(n\right)$ is the random part of the *n*th block, $c_{ij}$ is the correlation coefficient between two consecutive blocks, and $q_{ij}$ is a new random component, where $q_{ij}∼CN\left(0,1\right)$.

**Figure 2:**
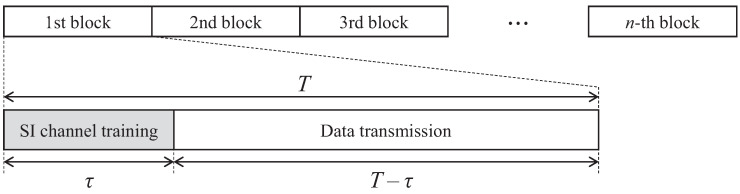
Frame structure for FD massive MIMO systems.

We considered a communication procedure that follows the frame structure illustrated in Figure 2. According to the channel model, the channels were assumed to be invariant during a coherent block of *T* channel uses. During a coherent block, τ channel uses are consumed for SI channel training, and the remaining (T−τ) channel uses are consumed for the FD data transmission of both the UL and DL. Without loss of generality, to study the effect of knowledge on the SI channel, it was assumed that the BS has perfect knowledge of $H^{u}$ and $H^{d}$ [17].

During the SI channel training phase, the BS transmits τ×1 pilot sequences $\psi_{m}$ to obtain ${\hat{h}}_{m}^{s}$, which is the estimate of the *m*th SI channel vector $h_{m}^{s}$. The pilot sequences are pairwise orthogonal, that is $\psi_{m}^{H}\psi_{m}=1$ and $\psi_{m}^{H}\psi_{m^{\prime }}=0$, where $m\neq m^{\prime }$.

After the SI channel training phase, the data transmission phase begins for FD transmission. The BS receives UL data from the $K_{u}$ UL UEs and transmits DL data to the $K_{d}$ DL UEs simultaneously. For the UL signal, the FD-BS conducts a series of signal processing procedures to eliminate the SI. In general, the FD receiver is composed of an analog SIC, analog-to-digital conversion (ADC), and digital SIC [14,15]. By passing through the FD receiver, the received UL signal is gradually restored. The received UL data signal vector for the $K_{u}$ UEs before SIC is given by:

(3) $$y_{u}=\underset{\mathrm{desired}\;\mathrm{signal}\;\mathrm{term}}{\underbrace{\sqrt{\rho_{u}}H^{u}x^{u}}}+\underset{\mathrm{SI}\;\mathrm{term}}{\underbrace{\sqrt{\rho_{s}}H^{s}Wx^{d}}}+z^{u},$$

where $x^{u}={\left[x_{1}^{u},\ldots ,x_{K_{u}}^{u}\right]}^{T}$ is a UL data signal vector, $\rho_{u}$ is the UL received power, $x^{d}={\left[x_{1}^{d},\ldots ,x_{K_{d}}^{d}\right]}^{T}$ is a DL data signal vector, $\rho_{s}$ is the DL power received at the BS (i.e., SI power), $W=\left[w_{1},\ldots ,w_{K_{d}}\right]$ with $\left|\right|w_{k}{\left|\right|}^{2}=1$ a DL precoding matrix, and $z^{u}={\left[z_{1}^{u},\ldots ,z_{M}^{u}\right]}^{T}$ is a Gaussian noise vector with zero mean and unit variance. After SIC, the received UL signal can be expressed as:

(4) $$\begin{array}{cc} y_{u,\mathrm{SIC}} & =y_{u}-\alpha \sqrt{\rho_{s}}{\hat{H}}^{s}Wx^{d}\\ \; & =\sqrt{\rho_{u}}H^{u}x^{u}+\underset{\mathrm{residual}\;\mathrm{SI}\;\mathrm{term}}{\underbrace{\alpha \sqrt{\rho_{s}}\left(H^{s}-{\hat{H}}^{s}\right)Wx^{d}}}+{\tilde{z}}^{u}, \end{array}$$

where ${\hat{H}}^{s}=\left[{\hat{h}}_{1}^{s},\ldots ,{\hat{h}}_{M}^{s}\right]$ is the estimated SI channel matrix, α is the analog SIC gain, and ${\tilde{z}}^{u}={\left[{\tilde{z}}_{1},\ldots ,{\tilde{z}}_{M}\right]}^{T}$ is the effective Gaussian noise, including the quantization error with zero mean and $\sigma_{\tilde{z}}^{2}$ variance. By applying the MIMO linear receiver to $y_{u,\mathrm{SIC}}$, we obtain the following:

(5) $$\begin{array}{cc} y_{u,\mathrm{rec}} & =G^{H}y_{u,\mathrm{SIC}}\\ \; & =\sqrt{\rho_{u}}G^{H}H^{u}x^{u}+\alpha \sqrt{\rho_{s}}G^{H}\left(H^{s}-{\hat{H}}^{s}\right)Wx^{d}+G^{H}{\tilde{z}}^{u}, \end{array}$$

where $G=\left[g_{1},\ldots ,g_{K_{u}}\right]$ with $\left|\right|g_{k}{\left|\right|}^{2}=1$ is the MIMO receiver matrix. Accordingly, the received UL signal for the *k*th UE is given by:

(6) $$\begin{array}{cc} r_{u,k} & =\sqrt{\rho_{u}}g_{k}^{H}h_{k}^{u}x_{k}^{u}+\sqrt{\rho_{u}}g_{k}^{H}\sum_{j\neq k}h_{j}^{u}x_{j}^{u}+\alpha \sqrt{\rho_{s}}g_{k}^{H}\left(H^{s}-{\hat{H}}^{s}\right)Wx^{d}+g_{k}^{H}{\tilde{z}}^{u}. \end{array}$$

The UL SINR for the *k*th UE is obtained by the following:

(7) $$\gamma_{u,k}=\frac{\rho_{u}{\left|g_{k}^{H}h_{k}^{u}\right|}^{2}}{\rho_{u}\sum_{j\neq k}{\left|g_{k}^{H}h_{j}^{u}\right|}^{2}+\alpha^{2}\rho_{s}{∥g_{k}^{H}\left(H^{s}-{\hat{H}}^{s}\right)W∥}^{2}+\sigma_{\tilde{z}}^{2}},$$

and the UL achievable rate for the *k*th UE is given by:

(8) $$R_{u,k}=\log_{2}\left(1+\gamma_{u,k}\right).$$

Meanwhile, the received DL data signal for the *k*th UE is given by:

(9) $$r_{d,k}=\sqrt{\rho_{d}}{\left(h_{k}^{d}\right)}^{H}w_{k}x_{k}+\sqrt{\rho_{d}}\sum_{j\neq k}{\left(h_{k}^{d}\right)}^{H}w_{j}x_{j}+z_{d,k},$$

where $\rho_{d}$ and $z_{d,k}$ are the DL power received at the *k*th UE and Gaussian noise with zero mean and unit variance, respectively. Thus, the DL SINR for the *k*th UE is obtained as follows:

(10) $$\gamma_{d,k}=\frac{\rho_{d}{\left|{\left(h_{k}^{d}\right)}^{H}w_{k}\right|}^{2}}{\rho_{d}\sum_{j\neq k}{\left|{\left(h_{k}^{d}\right)}^{H}w_{j}\right|}^{2}+1},$$

and the corresponding DL achievable rate is given by:

(11) $$R_{d,k}=\log_{2}\left(1+\gamma_{d,k}\right).$$

Finally, the total sum-rate reflecting the SI channel training overhead can be written as follows:

(12) $$\begin{array}{cc} \eta_{\mathrm{sum}} & =\eta_{u}+\eta_{d}\\ \; & =\left(1-\frac{\tau }{T}\right)\left(\sum_{k=1}^{K_{u}}R_{u,k}+\sum_{k=1}^{K_{d}}R_{d,k}\right). \end{array}$$

## 3. Proposed Self-Interference Channel Training

SIC performance at the FD receiver relies heavily on the accuracy of the SI channel estimation. To achieve a better SI channel estimation performance, a larger training overhead is required. However, increasing the training overhead for the SI channel reduces the duration of the data training phase, which can cause a large throughput loss for a large training overhead. The SI channel estimation issue is more problematic for massive MIMO systems because the training length for SI channel estimation based on orthogonal training linearly increases with the number of antennas [8]. For a massive MIMO, it is infeasible to employ full training overhead for SI channel estimation, where the training length is larger than or equal to the number of BS antennas. Therefore, we considered a more practical scenario for SI channel estimation at the FD-BS, where the training length is smaller than the number of BS antennas, that is τ<M. Considering the limited training overhead, we developed a partial SI channel training framework.

### 3.1. Partial Self-Interference Channel Training Framework

**Figure 3:**
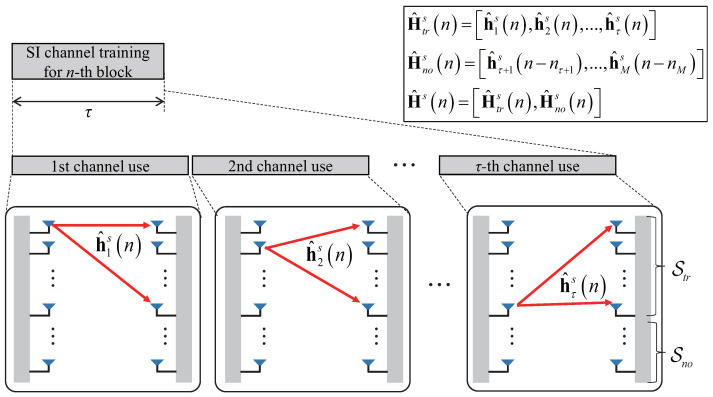
An example of partial SI channel training with limited training length, assuming τ=L for simplicity.

Figure 3 illustrates an example of the proposed partial SI channel training method for FD massive MIMO systems under a limited training overhead. Because τ<M at a given transmission block, it is possible to estimate at most τ SI channel vectors among the *M* vectors in the entire SI channel matrix. Let $S_{tr}\left(n\right)=\left\{i_{1},i_{2},\ldots ,i_{L}\right\}$ be the set of antenna indices for SI channel vectors estimated at the *n*th block and $S_{no}\left(n\right)=\left\{i_{1}^{\prime },i_{2}^{\prime },\ldots ,i_{M-L}^{\prime }\right\}$ be the set of antenna indices for SI channel vectors not estimated at the *n*th block, where the number of estimated SI channel vectors $L=\left|S_{tr}\left(n\right)\right|$ is no greater than τ because multiple training overhead can be used for a given SI channel vector. We can then write $H_{tr}^{s}\left(n\right)=\left[h_{i_{1}}\left(n\right),h_{i_{2}}\left(n\right),\ldots ,h_{i_{L}}\left(n\right)\right]$ as the M×L aggregated SI channel matrix to be estimated and $H_{no}^{s}\left(n\right)=\left[h_{i{}_{1}^{\prime }}\left(n\right),h_{i{}_{2}^{\prime }}\left(n\right),\ldots ,h_{i{}_{M-L}^{\prime }}\left(n\right)\right]$ as the remaining M×(M−L) aggregated SI channel matrix not to be estimated at the *n*th block. As shown in Figure 3, the BS consumes τ channel uses to estimate a set of SI channel vectors in $H_{tr}^{s}\left(n\right)$ by properly transmitting pilot signals. In contrast, the BS skips the channel estimation operation for the other remaining SI channel vectors included in $H_{no}^{s}\left(n\right)$. Instead, it is possible to reuse the last estimations of SI channel vectors obtained during the SI training phase in the previous transmission blocks.

To estimate $H_{tr}^{s}\left(n\right)$, the BS can transmit τ×L pilot sequences $\Psi =\left[\psi_{1},\ldots ,\psi_{L}\right]$, where $\Psi^{H}\Psi =I_{L}$. The M×τ signal matrix received during the training phase is then given by the following:

(13) $$Y_{tr}^{s}\left(n\right)=\sqrt{\rho_{p}}H_{tr}^{s}\left(n\right){P\Psi }^{H}+Z^{s},$$

where $\rho_{p}$ is the pilot power, $P=\mathrm{diag}\left(p_{1},\ldots ,p_{L}\right)$ is an L×L matrix representing the pilot allocation with $\sum_{i=1}^{L}p_{i}=\tau$, and $Z^{s}$ is an M×τ noise matrix with Gaussian elements of $CN\left(0,1\right)$. According to P, the BS can consume $p_{i}$ channel uses for transmitting $\psi_{i}$. From (13), the BS can obtain ${\hat{H}}_{tr}^{s}\left(n\right)$, which is the estimate of $H_{tr}^{s}\left(n\right)$, by applying the existing conventional linear estimator in the manner of the LS or minimum MSE (MMSE) [40,43]. For the other remaining SI channel vectors in $H_{no}^{s}\left(n\right)$, by using the nature of slowly-varying SI channels [29,30,31,32,33,34,35], the BS can reuse the previous estimated SI channel vectors, i.e.,

(14) $$\begin{array}{cc} {\hat{H}}_{no}^{s}\left(n\right) & =\left[{\hat{h}}_{i{}_{1}^{\prime }}^{s}\left(n\right),{\hat{h}}_{i{}_{2}^{\prime }}^{s}\left(n\right),\ldots ,{\hat{h}}_{i{}_{M-L}^{\prime }}^{s}\left(n\right)\right]\\ \; & =\left[{\hat{h}}_{i{}_{1}^{\prime }}^{s}\left(n-n_{i{}_{1}^{\prime }}\right),h_{i{}_{2}^{\prime }}^{s}\left(n-n_{i{}_{2}^{\prime }}\right),\ldots ,h_{i{}_{M-L}^{\prime }}^{s}\left(n-n_{i{}_{M-L}^{\prime }}\right)\right], \end{array}$$

where $n_{i}$ is the number of blocks passed after the last estimation of the *i*th SI channel vector. Consequently, the total estimated SI channel matrix at the *n*th block for the proposed partial training method is given as follows:

(15) $${\hat{H}}^{s}\left(n\right)=\mathrm{sort}\left\{\left[{\hat{H}}_{tr}^{s}\left(n\right),{\hat{H}}_{no}^{s}\left(n\right)\right]\right\}.$$

### 3.2. Problem Formulation

To further maximize the throughput, the set of SI channel vectors to be estimated at the given block should be appropriately selected. To formulate an optimization problem with explicit optimization parameters, we define an M×L selection matrix M, where the elements $m_{ij}=1$ if *i* is the *j*th element of $S_{tr}\left(n\right)$, and $m_{ij}=0$, otherwise. Then, (13) can be reformulated as:

(16) $$\begin{array}{cc} Y_{tr}^{s}\left(n\right) & =\sqrt{\rho_{p}}H^{s}\left(n\right)\mathrm{MP}\Psi^{H}+Z^{s}\\ \; & =\sqrt{\rho_{p}}H^{s}\left(n\right)\tilde{M}\Psi^{H}+Z^{s}, \end{array}$$

where $\tilde{M}\overset{\Delta }{\;=}\mathrm{MP}$ is an M×L matrix representing the antenna selection with a pilot allocation.

By considering the residual SI term in (4) as the metric, after removing the constant parameters α and $\sqrt{\rho_{s}}$, an optimization problem to minimize the expected residual SI power can be formulated as follows:

(17) $$\underset{\tilde{M}}{\arg\min E}\left[{∥G^{H}\left(n\right)\left(H^{s}\left(n\right)-{\hat{H}}^{s}\left(n\right)\right)W\left(n\right)x^{d}\left(n\right)∥}^{2}\right],$$

subject to $\sum_{i}\sum_{j}{\tilde{m}}_{ij}=\tau$.

To solve the optimization problem in (17), we analyzed the expected residual SI power and derived a closed-form expression of the expected residual SI power in terms of the SI channel estimation error as the following lemma.

**Lemma** **1.** Let $v_{ij}$ be the (i,j) element of $V=WW^{H}$, $u_{ij}$ be the (i,j) element of $U=GG^{H}$, and $\varepsilon_{ij}^{2}$ be the channel estimation error variance, defined as $\varepsilon_{ij}^{2}=E\left[\left(h_{ij}^{s}-{\hat{h}}_{ij}^{s}\right){\left(h_{ij}^{s}-{\hat{h}}_{ij}^{s}\right)}^{*}\right]$. The expected residual SI power is then given as follows:

(18) $$\begin{array}{c} E\left[{∥G^{H}\left(H^{s}-{\hat{H}}^{s}\right)Wx^{d}∥}^{2}\right]=\sum_{i=1}^{M}v_{ii}\cdot \left(\sum_{j=1}^{M}\varepsilon_{ij}^{2}u_{jj}\right). \end{array}$$

**Proof.**  See Appendix A. □

As shown in Lemma 1, the residual SI power is determined based on the coefficients of the DL precoder $v_{ii}$, the coefficients of the UL receiver $u_{jj}$, and the channel estimation error variance $\varepsilon_{ij}^{2}$. The BS already has knowledge of $v_{ii}$ and $u_{jj}$ for a given transmission block, and thus, $v_{ii}$ and $u_{jj}$ are deterministic. In contrast, $\varepsilon_{ij}^{2}$ varies depending on the SI channel training methodology. Given that a partial training strategy is employed, the channel estimation error variance for a given SI channel vector becomes different depending on whether the SI channel vector corresponds to the antenna in $S_{tr}$ or $S_{no}$. To quantify the estimation error variance, we derived the following lemma:

**Lemma** **2.** *Assuming the LS estimator, the SI channel estimation error variance at the nth transmission block is given by:*

(19) $$\varepsilon_{ij}^{2}\left(n\right)=\left\{\begin{array}{cc} \frac{1}{\sum_{j}{\tilde{m}}_{ij}\left(n\right)\cdot \rho_{p}} & \mathrm{for}\;i\in S_{tr}\left(n\right)\\ \frac{2-2\sqrt{{\left(c_{ij}\right)}^{n_{i}}}}{\kappa_{ij}+1}+\frac{1}{\sum_{j}{\tilde{m}}_{ij}\left(n-n_{i}\right)\cdot \rho_{p}} & \mathrm{for}\;i\in S_{no}\left(n\right) \end{array}\right..$$

**Proof.**  See Appendix B. □

**Figure 4:**
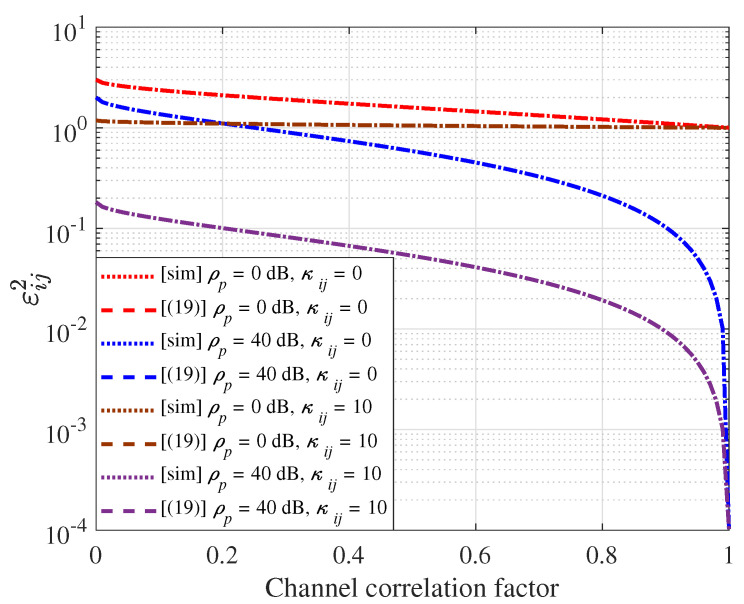
Comparison of the analyzed error variance in (19) and simulated values with M=64. The channel correlation factor along the x-axis corresponds to ${\left(c_{ij}\right)}^{n_{i}}$.

Figure 4 compares the simulation results for the error variance $\varepsilon_{ij}^{2}$ with Lemma 2 when M=64. It is shown that the analysis in Lemma 2 is correctly matched with the simulations. According to the results in Figure 4, it is shown that the estimation error variance decreases as the Rician factor increases because the effect of the deterministic part increases as the Rician factor increases. In addition, the estimation error variance decreases as the correlation factor increases. Therefore, we can conclude that our proposed SI channel estimation framework is a feasible solution for FD massive systems because the SI channel typically has a semi-static characteristic with large Rician and correlation factors.

Next, we reformulated the optimization problem to find an optimal pilot allocation ${\tilde{M}}^{*}$ by substituting (18) and (19) into (17) as:

(20) $$\underset{\tilde{M}\left(n\right)}{\arg\min}\sum_{i=1}^{M}v_{ii}\left(n\right)f\left(a_{i}\left(n\right),i\right),$$

where:

(21) $$f\left(a_{i}\left(n\right),i\right)=\left\{\begin{array}{cc} \sum_{j=1}^{M}\left(\frac{1}{a_{i}\left(n\right)\cdot \rho_{p}}\right)\cdot u_{jj}\left(n\right), & a_{i}\left(n\right)>0\\ \sum_{j=1}^{M}\left(\frac{2-2\sqrt{{\left(c_{ij}\right)}^{n_{i}}}}{\kappa_{ij}+1}+\frac{1}{a_{i}\left(n-n_{i}\right)\cdot \rho_{p}}\right)\cdot u_{jj}\left(n\right), & a_{i}\left(n\right)=0 \end{array}\right.$$

subject to $\sum_{i}a_{i}\left(n\right)=\tau$, where $a_{i}\left(n\right)=\sum_{j}{\tilde{m}}_{ij}\left(n\right)$ represents the number of allocated channel uses to estimate the *i*th SI channel vector.

### 3.3. Proposed Optimal SI Channel Training Strategy

The optimization problem in (20) is a mixed-integer nonlinear programming (MINLP) problem, which is generally difficult to solve [44]. To find an optimal solution of (20), an exhaustive search with a computational complexity of $O\left(2^{ML}\right)$ is required, which is infeasible considering a large *M* of massive MIMO. Therefore, as an alternative, we proposed a simple step-by-step algorithm to solve the optimization problem in (20), which is summarized as follows:

- Step 0 (preparation): Obtain V(n) and U(n) for the current transmission block. It is assumed that the BS already has knowledge of the Rician factors $\left(\kappa_{ij}\right)$, channel correlation coefficients $\left(c_{ij}\right)$, and the previous optimal pilot allocation strategy, that is ${\tilde{M}}^{*}\left(1\right),\ldots ,{\tilde{M}}^{*}\left(n-1\right)$.
- Step 1 (initialization): Initialize the parameter $a_{i}\left(n\right)=0$ for 1≤i≤M.
- Step 2 (calculation): Calculate the decrease in the residual SI power based on the objective function in (20) when the number of allocated channel uses increases by one, i.e.,
  $$\Delta_{i}\left(n\right)=v_{ii}\left(n\right)f\left(a_{i}\left(n\right),i\right)-v_{ii}\left(n\right)f\left(a_{i}\left(n\right)+1,i\right)$$
  for all antennas, 1≤i≤M. Find the antenna index that maximizes the decrement $\Delta_{i}\left(n\right)$, that is,
  (22) $$i^{*}=\underset{i}{\arg\max}\Delta_{i}\left(n\right).$$
  Increase the allocated channel uses for the $i^{*}$th antenna by:
  $$a_{i^{*}}\left(n\right)=a_{i^{*}}\left(n\right)+1.$$
- Step 3 (next allocation): Repeat Step 2 until $\sum_{i}a_{i}\left(n\right)=\tau$.

The proposed algorithm jointly finds the set of antenna indices to be estimated at the *n*th block and the number of channel uses for the corresponding pilot allocation. Owing to the closed-form expression of the objective function, it is possible to conjecture that the SI channel vector mostly contributes to the minimization of the residual SI power according to the pilot allocation without any trial on the real SI channel estimation. To obtain the initial sets of ${\tilde{M}}^{*}\left(1\right),\ldots ,{\tilde{M}}^{*}\left(n-1\right)$, the BS can simply estimate the SI channel vectors in a round-robin manner to ensure that the SI channel matrix has been estimated at least once before the *n*th block.

It is worth mentioning that the proposed algorithm was designed to find an optimal solution in a greedy manner for low complexity. In each iteration of the algorithm, it is ensured that there always exists only one solution $i^{*}$ to maximize $\Delta_{i}$. In addition, whenever an optimal $i^{*}$ is determined at a given iteration, the metric of the optimization problem in (20) decreases because $i^{*}$ is selected to reduce the metric based on (22). Consequently, the proposed algorithm always converges. Furthermore, the computational complexity of the proposed algorithm is dominated by the computational complexity of the matrix multiplication to obtain V(n) and U(n), that is $\max\left(O\left(M^{2}K_{d}\right),O\left(M^{2}K_{u}\right)\right)$. Therefore, although the proposed algorithm has a higher computational complexity compared with the simple random and round-robin training strategies, the proposed algorithm entails a significantly smaller computational complexity from an exhaustive search with a computational complexity of $O\left(2^{ML}\right)$.

## 4. Simulation Results

In this section, the results of the numerical evaluation are presented to verify the performance benefits of the proposed scheme. Random and round-robin training schemes were considered in addition to the proposed scheme. The BS antenna and pilot resource allocations were arbitrarily applied for every transmission block during random training, whereas the BS antenna was sequentially selected in a round-robin manner according to the selection of the last transmission block during the round-robin training. Furthermore, it was assumed that the BS has the outdated SI channel information before the initial transmission block. The LS channel estimator was considered for the SI channel estimation. Further, the zero-forcing (ZF) beamformer was employed for DL transmission, whereas the ZF receiver was employed for UL reception. In addition, unless specified otherwise, the following parameters were considered: M=64, T=128, $\rho_{p}=40$ dB, $\rho_{d}=30$ dB, $\rho_{u}=15$ dB, $c_{ij}=0.9$, and 10 bit quantization for ADC [17,41,45]. Finally, considering the limited resources for the training overhead, the maximum τ was set to 48 among the T(=128) channel uses for each transmission block [46].

**Figure 5:**
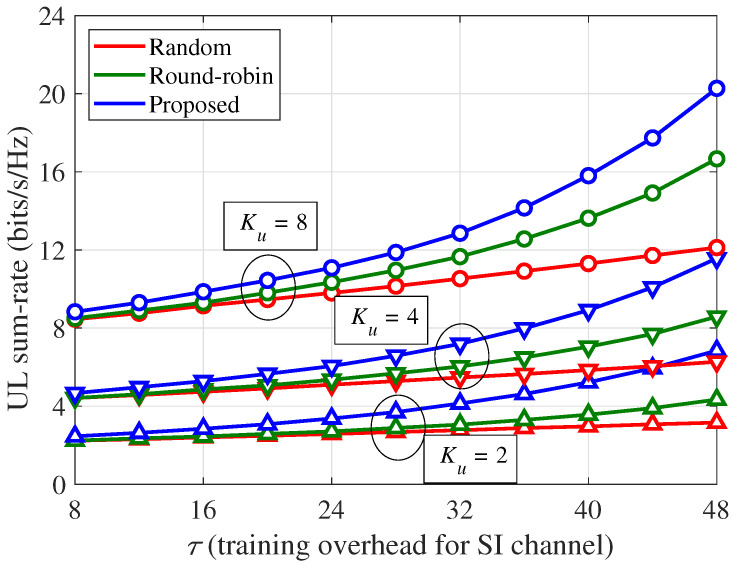
UL sum-rate ($\sum_{k}R_{u,k}$) per channel use according to the number of UEs ($K_{u}$) and SI training overhead (τ): α=70 dB, $\kappa_{ij}=3$, and 80 dB path loss between the BS and UE.

Figure 5 shows the UL sum-rate ($\sum_{k}R_{u,k}$) of the training strategies per channel use according to the number of UEs ($K_{u}$) and the SI training overhead (τ), where α=70 dB [47,48] and $\kappa_{ij}=3$ [38], and an 80 dB path loss (obtained by assuming a 100 m distance between the BS and UE with a path loss exponent of four [17]) between the BS and UE was considered. The UL sum-rates per channel use in Figure 5 are not normalized by the amount of training overhead τ, and the SI channel estimation accuracy improves regardless of the training strategy as τ increases. Therefore, the UL sum-rates per channel use in Figure 5 improve regardless of the training strategy as τ increases. Nevertheless, by considering the pilot resource allocation to minimize the residual SI power, the proposed scheme outperforms the random and round-robin schemes. Furthermore, because the minimization of the residual SI power can be more effectively performed, the performance gap of the proposed scheme over the other schemes increases with τ. Specifically, when τ=48, the UL sum-rate increments of the proposed scheme over the random training are approximately 3.69, 5.29, and 8.15 bits/s/Hz for $K_{u}=$ 2, 4, and 8, respectively, whereas those of the proposed scheme over the round-robin training are approximately 2.51, 2.98, and 3.61 bits/s/Hz for $K_{u}=$ 2, 4, and 8, respectively.

**Figure 6:**
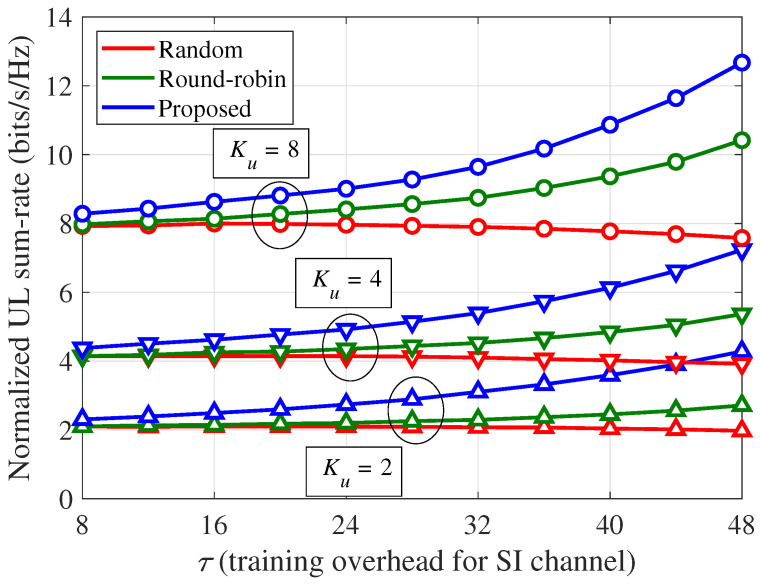
Normalized UL sum-rate ($\eta_{u}$) per channel use according to the number of UEs ($K_{u}$) and SI training overhead (τ): α=70 dB, $\kappa_{ij}=3$, and 80 dB path loss between the BS and UE.

Figure 6 shows the normalized UL sum-rate ($\eta_{u}$) of the training strategies per channel use according to the number of UEs ($K_{u}$) and the SI training overhead (τ), where the system parameters are identical to those in Figure 5. Unlike the results in Figure 5, the UL sum-rate of the random training decreases as τ increases because of the arbitrary pilot allocation in each transmission block. Meanwhile, as the estimation of each SI channel vector is sequentially applied in each block, the round-robin training can prioritize the update of more outdated estimated SI channel vectors, which results in a gradual performance improvement for a larger τ. In contrast, for the given τ and $K_{u}$, the proposed scheme outperforms the round-robin training, as well as the random training, which implies that the pilot resource allocations for the minimization of the residual SI power are more effective than those for an update of more outdated SI channel information. When τ=48, the normalized UL sum-rate improvements of the proposed scheme over the random training are approximately 116.7%, 84.7%, and 67.3% for $K_{u}=$ 2, 4, and 8, respectively, and those of the proposed scheme over the round-robin training are approximately 57.8%, 34.7%, and 21.7% for $K_{u}=$ 2, 4, and 8, respectively.

**Figure 7:**
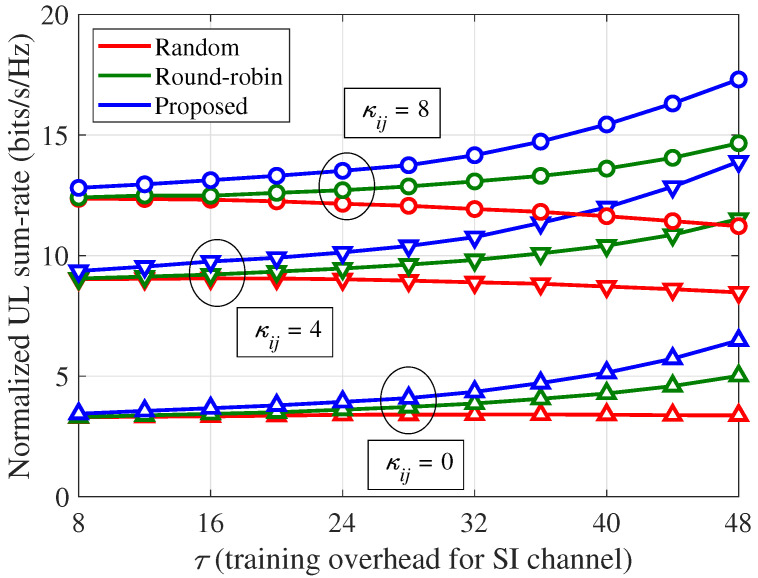
Normalized UL sum-rate ($\eta_{u}$) per channel use according to the Rician factor ($\kappa_{ij}$) and SI training overhead (τ): $K_{u}=8$, α=70 dB, and 80 dB path loss between the BS and UE.

In Figure 7, the effects of the Rician factor ($\kappa_{ij}$) on the normalized UL sum-rate of the partial training strategy are shown, where $K_{u}=8$, α=70 dB, and 80 dB path loss between the BS and UE are considered. Because the SI channel becomes more deterministic as $\kappa_{ij}$ increases, the utilization of the previous estimates yields fewer errors, which leads to a performance improvement of the partial training strategy for a larger $\kappa_{ij}$. Meanwhile, as $\kappa_{ij}$ decreases, the residual SI power can be significantly affected by the pilot resource allocations, and the performance improvement ratio of the proposed scheme over the other schemes can be increased for a smaller $\kappa_{ij}$. Specifically, when τ=48, the normalized UL sum-rate improvements of the proposed scheme over the random training are approximately 92.1%, 64.0%, and 54.2% for $\kappa_{ij}=$ 0, 4, and 8, respectively, and those of the proposed scheme over the round-robin training are approximately 29.4%, 20.7%, and 18.1% for $\kappa_{ij}=$ 0, 4, and 8, respectively.

**Figure 8:**
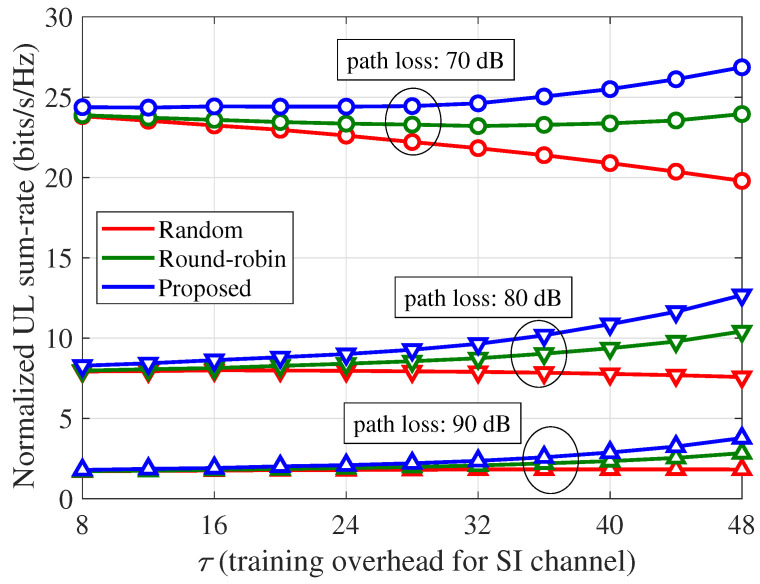
Normalized UL sum-rate ($\eta_{u}$) per channel use according to the path loss between the BS and UE and SI training overhead (τ): $K_{u}=8$, α=70 dB, and $\kappa_{ij}=3$.

In Figure 8, the effects of the path loss between the BS and UE on the normalized UL sum-rate of the partial training strategy are illustrated, where $K_{u}=8$, α=70 dB, and $\kappa_{ij}=3$. As the path loss increases, more DL power is required to achieve the same $\rho_{d}$, which results in severe SI problems owing to the increased SI power. Therefore, as the path loss increases, the UL sum-rates of FD massive MIMO systems are significantly degraded, although the minimization of the residual SI power becomes more important. Therefore, the normalized UL sum-rate improvement ratios of the proposed scheme over the other schemes are more significant as the path loss increases.

**Figure 9:**
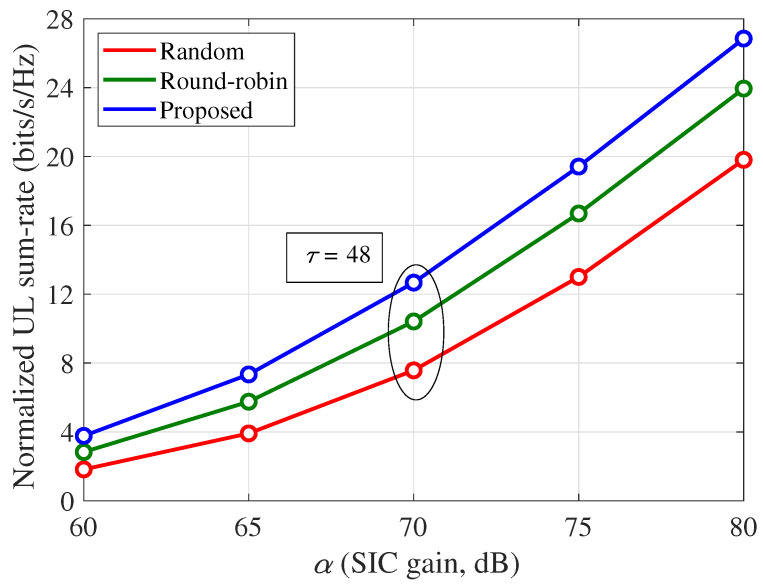
Normalized UL sum-rate ($\eta_{u}$) per channel use according to the analog SIC gain (α): $K_{u}=8$, $\kappa_{ij}=3$, τ=48, and 80 dB path loss between the BS and UE.

Figure 9 shows the normalized UL sum-rates per channel use according to the analog SIC gain (α), where $K_{u}=8$, $\kappa_{ij}=3$, τ=48, and 80 dB path loss between the BS and UE is considered. It is shown that the proposed scheme obtains better UL sum-rates than the other schemes regardless of α. The performance improvement ratios of the proposed scheme increase for a smaller α, because a smaller α indicates more severe SI problems at the FD-BS, similar to the case of the larger path loss shown in Figure 8.

The results in Figure 5, Figure 6, Figure 7, Figure 8 and Figure 9 show that the proposed scheme can achieve a larger UL sum-rate for a given SI channel training length compared to the other training schemes. This implies that, to achieve a target UL sum-rate, a smaller training overhead for the SI channel is required for the proposed scheme compared to the other training schemes. Because the proposed scheme requires a smaller training overhead to estimate the SI channel for a given target UL sum-rate, by having a larger data transmission phase, the proposed scheme can also achieve a larger normalized DL sum-rate than the other training schemes.

**Figure 10:**
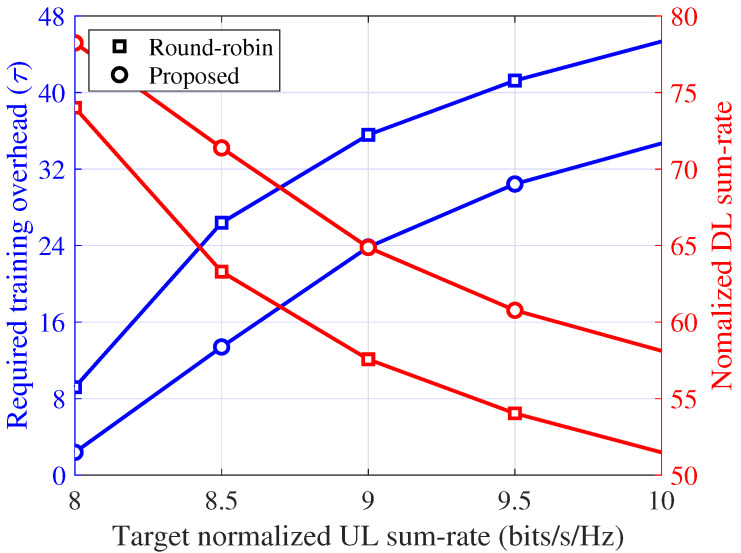
Required training overhead (τ) and normalized DL sum-rate ($\eta_{d}$) per channel use according to the target normalized UL sum-rate: $K_{u}=8$, $\kappa_{ij}=3$, and 80 dB path loss between the BS and UE.

Therefore, in Figure 10, the required training overhead and corresponding normalized DL sum-rate according to the target normalized UL sum-rate are shown for the proposed scheme and round-robin training. The results for the random training were omitted because they cannot meet the target normalized UL sum-rate of interest. As shown in Figure 10, to achieve a given target normalized UL sum-rate, the proposed scheme requires approximately 26.1% to 76.2% smaller training overhead (τ) compared with the round-robin training. That is, the portion of the data transmission phase (1−τ/T) in each symbol block can also be increased by the proposed scheme. As a result, for a given target normalized UL sum-rate, the proposed scheme can achieve a maximum improvement of approximately 13.2% on the normalized DL sum-rate from the round-robin training.

**Figure 11:**
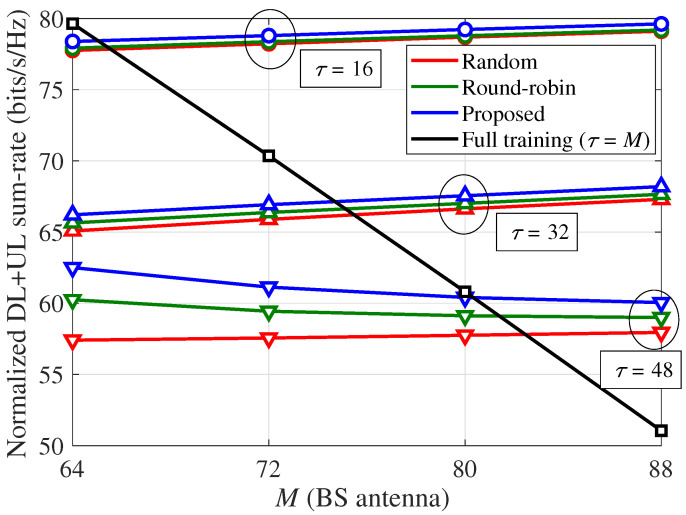
Normalized DL and UL sum-rate ($\eta_{\mathrm{sum}}$) per channel use according to the BS antenna (*M*): $K_{u}=8$, α=70 dB, $\kappa_{ij}=3$, and 80 dB path loss between the BS and UE.

Finally, in Figure 11, the normalized DL and UL sum-rate ($\eta_{\mathrm{sum}}$) of the partial training strategies per channel use according to the number of BS antennas (*M*) is compared with that of the full training strategy. Because the full training requires τ=M to estimate all SI channel vectors, the portion of the data transmission phase, which is used for the full-duplex data transmission of both DL and UL, in each symbol block decreases as *M* increases. As a result, the normalized sum-rate of full training rapidly decreases for a larger *M* and approaches zero when M=T. In contrast, in cases of partial training strategies, the portion of the data transmission phase decreases as τ increases instead of *M* in full training. Therefore, although the normalized UL sum-rate of the partial training strategies increases with τ for a given *M* and *T* as shown in Figure 5, Figure 6, Figure 7, Figure 8 and Figure 9, the normalized DL and UL sum-rate of the partial training strategies can be decreased for a larger τ. This implies that the selection of τ for the proposed scheme should be decided based on the target service application with a given requirement for the UL and DL rates.

## 5. Conclusions

In this study, we investigated a novel SI channel training framework for FD massive MIMO systems with limited training overhead. The proposed scheme enables a partial training strategy to minimize the expected residual SI power, and it is shown that the objective function of the optimization problem can be reformulated in terms of the parameters of antenna selection and pilot resource allocations. From numerical evaluations, it was verified that the proposed scheme is an effective SI channel training strategy for FD massive MIMO systems, particularly when the system suffers from severe SI signals. In this study, it was assumed that the training overhead and pilot power are already provided by the system. Thus, the proposed scheme can be extended to jointly optimize the training overhead and pilot power with antenna selection and pilot resource allocation. This remains within the scope of future studies.

## Data Availability

Not applicable.

## Appendix A. Proof of Lemma 1

By defining $E\overset{\Delta }{\;=}H^{s}-{\hat{H}}^{s}$, the expected residual SI power is calculated as follows:

(A1) $$\begin{array}{cc} E\left[{∥G^{H}EWx^{d}∥}^{2}\right] & =E\left[\mathrm{tr}\left({\left(x^{d}\right)}^{H}W^{H}E^{H}GG^{H}EWx^{d}\right)\right]\\ \; & =E\left[\mathrm{tr}\left(Wx^{d}{\left(x^{d}\right)}^{H}W^{H}E^{H}GG^{H}E\right)\right]\\ \; & =\mathrm{tr}\left(E\left[W\right]E\left[x^{d}{\left(x^{d}\right)}^{H}\right]E\left[W^{H}\right]E\left[E^{H}GG^{H}E\right]\right)\\ \; & \overset{\left(a\right)}{=}\mathrm{tr}\left(WW^{H}E\left[E^{H}{\mathrm{GG}}^{H}E\right]\right), \end{array}$$

where (*a*) is from $E\left[x^{d}{\left(x^{d}\right)}^{H}\right]=I_{K_{d}}$. Let $U\overset{\Delta }{\;=}GG^{H}$, and the term $E\left[E^{H}UE\right]$ in (A1) can then be formulated as follows:

(A2) $$\begin{array}{cc} E\left[E^{H}UE\right] & =E\left[\left[\begin{array}{c} \varepsilon_{1}^{H}\\ \vdots \\ \varepsilon_{M}^{H} \end{array}\right]U\left[\begin{array}{ccc} \varepsilon_{1} & \cdots & \varepsilon_{M} \end{array}\right]\right]\\ \; & =E\left[\begin{array}{ccc} \varepsilon_{1}^{H}U\varepsilon_{1} & \cdots & \varepsilon_{1}^{H}U\varepsilon_{M}\\ \vdots & \ddots & \vdots \\ \varepsilon_{M}^{H}U\varepsilon_{1} & \ldots & \varepsilon_{M}^{H}U\varepsilon_{M} \end{array}\right]\\ \; & \overset{\left(b\right)}{=}E\left[\begin{array}{ccc} \varepsilon_{1}^{H}U\varepsilon_{1} & \cdots & 0\\ \vdots & \ddots & \vdots \\ 0 & \ldots & \varepsilon_{M}^{H}U\varepsilon_{M} \end{array}\right], \end{array}$$

where (*b*) is derived from the fact that the estimation errors for the different channel vectors are uncorrelated. Here, $E\left[\varepsilon_{i}^{H}U\varepsilon_{i}\right]$ for 1≤i≤M can be calculated as follows:

(A3) $$\begin{array}{cc} E\left[\varepsilon_{i}^{H}U\varepsilon_{i}\right] & =E\left[\mathrm{tr}\left(\varepsilon_{i}^{H}U\varepsilon_{i}\right)\right]\\ \; & =E\left[\mathrm{tr}\left(\varepsilon_{i}\varepsilon_{i}^{H}U\right)\right]\\ \; & =\mathrm{tr}\left(E\left[\varepsilon_{i}\varepsilon_{i}^{H}\right]U\right)\\ \; & =\mathrm{tr}\left(E\left[\begin{array}{ccc} \varepsilon_{i1}\varepsilon_{i1}^{*} & \cdots & \varepsilon_{i1}\varepsilon_{iM}^{*}\\ \vdots & \ddots & \vdots \\ \varepsilon_{iM}\varepsilon_{i1}^{*} & \cdots & \varepsilon_{iM}\varepsilon_{iM}^{*} \end{array}\right]U\right)\\ \; & \overset{\left(c\right)}{=}\mathrm{tr}\left(E\left[\begin{array}{ccc} \varepsilon_{i1}\varepsilon_{i1}^{*} & \cdots & 0\\ \vdots & \ddots & \vdots \\ 0 & \cdots & \varepsilon_{iM}\varepsilon_{iM}^{*} \end{array}\right]U\right)\\ \; & =\sum_{j=1}^{M}\varepsilon_{ij}^{2}u_{jj}, \end{array}$$

where (*c*) is derived from the fact that the estimation errors for different channel elements are uncorrelated. By substituting (A2) and (A3) into (A1), we obtain Lemma 1.

## Appendix B. Proof of Lemma 2

For $i\in S_{tr}\left(n\right)$, the *i*th SI channel vector for the *n*th block is estimated based on the LS estimation. In this case, the channel estimation error variance $\varepsilon_{ij}^{2}$ is given by the following [43]:

(A4) $$\varepsilon_{ij}^{2}=\frac{1}{a_{i}\left(n\right)\rho_{p}},$$

where $a_{i}\left(n\right)=\sum_{j}{\tilde{m}}_{ij}\left(n\right)$ is the number of channel uses consumed for transmitting the pilot signal to estimate the *i*th SI channel vector.

For $i\in S_{tr}\left(n\right)$, the BS skips the estimation of the *i*th SI channel vector for the *n*th block. Instead, the previously estimated *i*th SI channel vector, that is ${\hat{h}}_{i}^{s}\left(n-n_{i}\right)$, replaces ${\hat{h}}_{i}^{s}\left(n\right)$. Therefore, the error variance is defined as follows:

(A5) $$\varepsilon_{ij}^{2}\left(n\right)=E\left[{\left(h_{ij}^{s}\left(n\right)-{\hat{h}}_{ij}^{s}\left(n-n_{i}\right)\right)}^{*}\left(h_{ij}^{s}\left(n\right)-{\hat{h}}_{ij}^{s}\left(n-n_{i}\right)\right)\right].$$

By substituting the channel characteristics defined in (1) and (2) into (A5), we obtain:

(A6) $$\begin{array}{cc} \; & E\left[{\left(h_{ij}^{s}\left(n\right)-{\hat{h}}_{ij}^{s}\left(n-n_{i}\right)\right)}^{*}\left(h_{ij}^{s}\left(n\right)-{\hat{h}}_{ij}^{s}\left(n-n_{i}\right)\right)\right]\\ \; & =\frac{\left(1-{\left(c_{ij}\right)}^{n_{i}}\right)}{\kappa_{ij}+1}E\left[q_{ij}^{*}q_{ij}\right]\\ \; & +E\left[\begin{array}{c} {\left(h_{ij}^{s}\left(n-n_{i}\right)-{\hat{h}}_{ij}^{s}\left(n-n_{i}\right)+\sqrt{\frac{1}{\kappa_{ij}+1}}\left(\sqrt{{\left(c_{ij}\right)}^{n_{i}}}-1\right){\tilde{h}}_{ij}^{s}\left(n-n_{i}\right)\right)}^{*}\\ \times (h_{ij}^{s}\left(n-n_{i}\right)-{\hat{h}}_{ij}^{s}\left(n-n_{i}\right)+\sqrt{\frac{1}{\kappa_{ij}+1}}\left(\sqrt{{\left(c_{ij}\right)}^{n_{i}}}-1\right){\tilde{h}}_{ij}^{s}\left(n-n_{i}\right)) \end{array}\right]\\ \; & =\frac{\left(1-{\left(c_{ij}\right)}^{n_{i}}\right)}{\kappa_{ij}+1}+E\left[{\left(h_{ij}^{s}\left(n-n_{i}\right)-{\hat{h}}_{ij}^{s}\left(n-n_{i}\right)\right)}^{*}(h_{ij}^{s}\left(n-n_{i}\right)-{\hat{h}}_{ij}^{s}\left(n-n_{i}\right))\right]\\ \; & +\frac{{\left(\sqrt{{\left(c_{ij}\right)}^{n_{i}}}-1\right)}^{2}}{\kappa_{ij}+1}E\left[{\left({\tilde{h}}_{ij}^{s}\right)}^{*}\left(n-n_{i}\right){\tilde{h}}_{ij}^{s}\left(n-n_{i}\right)\right]\\ \; & +\sqrt{\frac{1}{\kappa_{ij}+1}}\left(\sqrt{{\left(c_{ij}\right)}^{n_{i}}}-1\right)\left(\begin{array}{c} E\left[{\left(h_{ij}^{s}\left(n-n_{i}\right)-{\hat{h}}_{ij}^{s}\left(n-n_{i}\right)\right)}^{*}{\tilde{h}}_{ij}^{s}\left(n-n_{i}\right)\right]\\ +E\left[{\left({\tilde{h}}_{ij}^{s}\right)}^{*}\left(n-n_{i}\right)(h_{ij}^{s}\left(n-n_{i}\right)-{\hat{h}}_{ij}^{s}\left(n-n_{i}\right))\right] \end{array}\right)\\ \; & \overset{\left(a\right)}{=}\frac{\left(1-{\left(c_{ij}\right)}^{n_{i}}\right)}{\kappa_{ij}+1}+\frac{1}{a_{i}\left(n-n_{i}\right)\cdot \rho_{p}}+\frac{{\left(\sqrt{{\left(c_{ij}\right)}^{n_{i}}}-1\right)}^{2}}{\kappa_{ij}+1}\\ \; & +\sqrt{\frac{1}{\kappa_{ij}+1}}\left(\sqrt{{\left(c_{ij}\right)}^{n_{i}}}-1\right)\left(E\left[{\left(\delta_{ij}\left(n-n_{i}\right)\right)}^{*}{\tilde{h}}_{ij}^{s}\left(n-n_{i}\right)\right]+E\left[{\left({\tilde{h}}_{ij}^{s}\right)}^{*}\left(n-n_{i}\right)\delta_{ij}\left(n-n_{i}\right)\right]\right)\\ \; & \overset{\left(b\right)}{=}\frac{\left(1-{\left(c_{ij}\right)}^{n_{i}}\right)}{\kappa_{ij}+1}+\frac{1}{a_{i}\left(n-n_{i}\right)\cdot \rho_{p}}+\frac{{\left(\sqrt{{\left(c_{ij}\right)}^{n_{i}}}-1\right)}^{2}}{\kappa_{ij}+1}\\ \; & =\frac{2-2\sqrt{{\left(c_{ij}\right)}^{n_{i}}}}{\kappa_{ij}+1}+\frac{1}{a_{i}\left(n-n_{i}\right)\cdot \rho_{p}}. \end{array}$$

In (A6), (*a*) is derived from $E\left[{\left(h_{ij}^{s}\left(n-n_{i}\right)-{\hat{h}}_{ij}^{s}\left(n-n_{i}\right)\right)}^{*}\left(h_{ij}^{s}\left(n-n_{i}\right)-{\hat{h}}_{ij}^{s}\left(n-n_{i}\right)\right)\right]=\frac{1}{a_{i}\left(n-n_{i}\right)\cdot \rho_{p}}$ and $E\left[{\left({\tilde{h}}_{ij}^{s}\right)}^{*}\left(n-n_{i}\right){\tilde{h}}_{ij}^{s}\left(n-n_{i}\right)\right]=1$, and (*b*) is derived from $E\left[\delta_{ij}\left(n-n_{i}\right)\right]=0$ with $\delta_{ij}\left(n-n_{i}\right)\overset{\Delta }{\;=}h_{ij}^{s}\left(n-n_{i}\right)-{\hat{h}}_{ij}^{s}\left(n-n_{i}\right)$. Consequently, we obtain Lemma 2.
